# Blind identification of the spinal cord output in humans with high-density electrode arrays implanted in muscles

**DOI:** 10.1126/sciadv.abo5040

**Published:** 2022-11-16

**Authors:** Silvia Muceli, Wigand Poppendieck, Aleš Holobar, Simon Gandevia, David Liebetanz, Dario Farina

**Affiliations:** ^1^ Department of Electrical Engineering, Chalmers University of Technology, Gothenburg, Sweden.; ^2^ Mannheim University of Applied Sciences, Mannheim, Germany.; ^3^ Faculty of Electrical Engineering and Computer Science, University of Maribor, Maribor, Slovenia.; ^4^ Neuroscience Research Australia and University of New South Wales, Randwick, Sydney, New South Wales, Australia.; ^5^ Department of Neurology, University Medical Center Göttingen, Georg-August University, Göttingen, Germany.; ^6^ Department of Bioengineering, Imperial College London, London, UK.

## Abstract

Invasive electromyography opened a new window to explore motoneuron behavior in vivo. However, the technique is limited by the small fraction of active motoneurons that can be concurrently detected, precluding a population analysis in natural tasks. Here, we developed a high-density intramuscular electrode for in vivo human recordings along with a fully automatic methodology that could detect the discharges of action potentials of up to 67 concurrently active motoneurons with 99% accuracy. These data revealed that motoneurons of the same pool receive common synaptic input at frequencies up to 75 Hz and that late-recruited motoneurons inhibit the discharges of those recruited earlier. These results constitute an important step in the population coding analysis of the human motor system in vivo.

## Teaser

Developing implanted electrodes and an automatic decomposition algorithm addresses open questions in motoneuron physiology.

## INTRODUCTION

The introduction of intramuscular needles and wires for electromyography (EMG) by Adrian and Bronk (*1*) and Basmajian and Stecko (*2*) opened a window to explore the neural underpinning of movement control. By recording muscle fiber action potentials, intramuscular EMG reveals the timing of the action potentials discharged by the innervating spinal motoneurons (MNs). The analysis of motor units (MUs) from decomposition of intramuscular EMG signals recorded with needle and fine wire electrodes rapidly became the standard approach to study MN behavior in vivo in humans and other species (*3*).

Nonetheless, the use of EMG to assess MNs also imposes some constraints. Some intramuscular electrodes are highly selective to detect the electrical activity of a small number of muscle fibers. This makes it easy to identify the discharge times of a few MUs through EMG decomposition, which is conventionally based on spike sorting of action potentials with similar morphology (*4*). However, the electrode selectivity implies that only a small fraction of the hundreds of active MNs can be studied concurrently. To increase the number of sampled MUs, investigators have serially recorded single MU activity. While serial recordings have unraveled patterns of MN discharges, a MN population analysis is still missing, which limits our understanding of the process of generation of the neural output of the spinal cord. Now, there is no robust method that provides simultaneous decoding of a large portion of the active MNs in natural tasks.

The identification of large populations of concurrently active MUs is necessary to characterize the synaptic inputs received by MNs. Coherence among discharge patterns of the homonymous MN pool reflects the common synaptic input at various frequency bands. The expression “discharge pattern” is used in this study to indicate the series of discharge times of each MU. Note that this terminology does not imply stationarity of the time series of the MU discharge times. A single MN cannot accurately sample an input with a frequency greater than half its average discharge rate (*5*, *6*), which is usually in the range of 10 to 40 Hz (*7*). As a result, sampling by few MNs limits the frequency range at which coherence (and thus common synaptic input) can be observed. However, as the common synaptic input is spread to the whole MN pool (*8*), pooling the discharge patterns extracted from large populations of MUs allows sampling at higher frequencies.

As a further example, analysis of the output of a population of MNs is also a way to investigate connectivity among MNs, e.g., due to Renshaw inhibition (*9*, *10*). Renshaw cells receive collateral projections from MN axons and synapse on MNs mediating recurrent inhibition back to the MN pool. However, the distribution of recurrent inhibition throughout the MN pool is unknown in humans (*11*). Most knowledge about recurrent inhibition stems from experiments on anesthetized animal preparations, and direct translation of findings to human studies of intact MNs during natural behavior is challenging. Again, technological advances for sampling large populations of MUs in vivo in humans are necessary (*11*).

A way to increase the number of concurrently detected MUs in natural tasks uses decomposition of activity recorded with high-density grids of surface electrodes (*12*). However, surface EMG only detects the activity of superficial MUs (*13*). As an alternative approach to increase the number of sampled MUs, we previously introduced multichannel intramuscular electrodes based on thin-film technology (*14*, *15*), which provide a large and unbiased sample of MUs from both deep and superficial muscles. These electrodes comprise a linear array of detection points in a flexible wire that can record across the muscle cross section. Tens of MUs can be concurrently detected with these systems (*15*). However, these systems are limited to only 16 electrode sites, and they require partially manual spike sorting. Spike sorting software for multichannel intramuscular EMG now relies on human oversight to edit the results (*16*).

When increasing the number of recorded signals, the EMG decomposition process must be applied to each recorded EMG channel. With conventional spike sorting, this increases computation time and manual editing of the results (*17*). Alternative to spike sorting, blind source separation (BSS) methods can be applied to separate sources (i.e., to decompose EMG signals into the constituent trains of MU action potentials) when a large number of observations (EMG channels) are available (*18*). However, classic BSS limits the maximum number of extracted sources to the number of observations (in practice to less than the observations).

Here, we describe two breakthroughs in the technology to investigate MN behavior in vivo. First, we designed, manufactured, and tested a novel implantable electrode array for human studies with a much greater number of recording sites and higher site density than any previous systems. The novel design allowed the implantation of the array acutely with needles of similar size to those used in conventional concentric needle recording. Second, we used a fully automatic decomposition algorithm (no manual editing) that enabled the decoding of the high-density multiunit recordings with accuracy comparable to that achieved by extensive manual editing of each trace by an expert operator. Furthermore, with these new technologies, we addressed two fundamental open questions in MN physiology. We found that a MN pool receives common synaptic input in a frequency range up to 75 Hz, much greater than previously thought (*19*). We then analyzed the effect of individual MU discharges on the MN population output to determine the connectivity among MNs.

## RESULTS

### Intramuscular thin-film electrode array

**Fig. 1.**
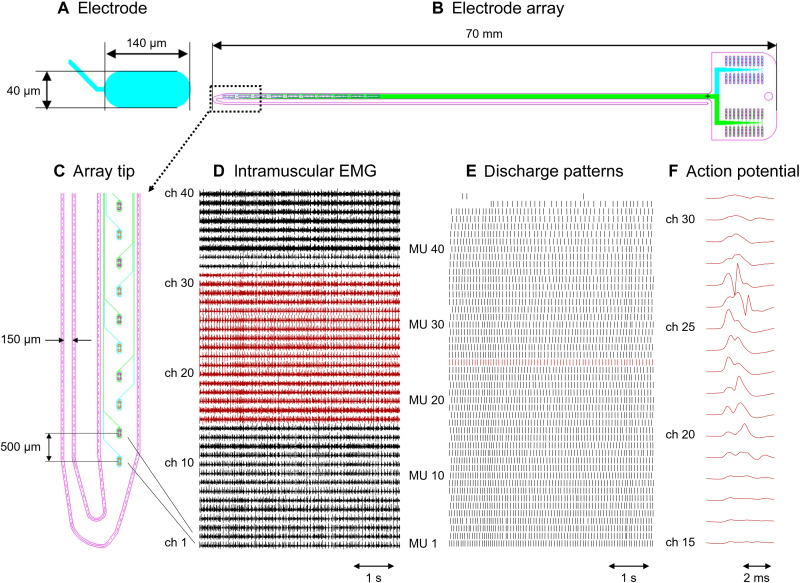
Design of the double-sided electrode array and representative recordings. (**A**) Close-up of an oval electrode. (**B**) Whole structures with the tracks running toward the connection pad. (**C**) Close-up of the electrode array tip. Electrodes represented in cyan are located on the top side of the thin-film array, and those in green are located on the bottom side of the wider filament. (**D**) Representative recordings obtained from the tibialis anterior of subject 1 (S1) during a contraction at 20% of the maximal force [maximal voluntary contraction (MVC)]. (**E**) Discharge pattern of 47 motor units (MUs) extracted from the signal shown in (D). (**F**) Multichannel action potentials of a representative MU obtained by averaging the red-colored EMG channels in (D) with the discharge pattern of the same color in (E) as a trigger. ch, channel.

We designed and manufactured a high-density intramuscular array with 40 platinum electrodes with an area of 5257 μm^2^ each (Fig. 1A), linearly distributed over a length of 2 cm. Figure 1B shows the complete layout of the double-sided thin-film structure. The structure is built on a polyimide substrate, has a total length of 7 cm, and is U-shaped with two filaments with a width of 655 and 150 μm (Fig. 1C) and a thickness of 20 μm. The wider filament contains two linear arrays of 20 oval electrodes each (Fig. 1A), with a 1-mm interelectrode distance on the top (cyan) and bottom (green) sides of the polyimide (Fig. 1C). The two arrays have a shift of 0.5 mm (Fig. 1C). Because the double-sided structure is only 20 μm thick, it is equivalent to a linear array of electrodes with an interelectrode distance of 0.5 mm. The number of electrodes is limited by the number of interconnection lines fitting on the filament. The advantage of two arrays on the two sides of the structure is that the filament width can be reduced for a given number of electrodes. In addition, the occurrence of short circuits during manufacturing is reduced. The narrower filament is inserted into a 25-gauge needle (100 Sterican, B. Braun, Melsungen, Germany) to introduce the thin-film structure into a muscle, with a procedure similar to that used in classic fine wire EMG. The needle is withdrawn, leaving the array inside the muscle.

### Signal quality and MU yield

The electrode array was tested in three healthy men [subject 1 (S1) to subject 3 (S3)]. Two arrays were inserted in the tibialis anterior of subject S1, while one array was implanted in the other two subjects. S1 performed a steady contraction at 20% of maximal voluntary contraction (MVC), whereas subject 2 (S2) and S3 contracted the tibialis at 30% MVC. The electrodes recorded high-quality signals, with a baseline noise of 15.8 ± 9.9 μV (average ± SD across four arrays of 40 channels each). Figure 1D displays representative signals recorded from S1 to show the signal-to-noise ratio. Figure 1E shows the discharge patterns of the MUs extracted via manual decomposition from the signals recorded from array 1 in S1. In the raster plot, each row represents a different MU, and each vertical line represents the discharge time of an action potential. Within the selected time frame (5 s), 45 MUs were consistently detected as active, 1 MU was recruited during the contraction, and 1 MU had a few isolated discharges. Figure 1F shows a representative example of a MU action potential detected across several electrodes of the 40-channel array.

The recorded signals were decomposed independently into the constituent MU discharge patterns by two expert investigators (S.M. and A.H.) using two decomposition approaches. We refer to them as manual and automatic decomposition. For manual decomposition, intramuscular EMG signals from each thin-film system were decomposed channel by channel using spike sorting software (*16*) and manually edited for resolving missed discharges and superimpositions. As the territory of each MU [i.e., the anatomical cross-sectional area occupied by the muscle fibers belonging to the same MU (*20*, *21*)] extended over multiple channels, the discharge pattern of a certain MU was identified from different channels (see the next paragraph). This redundant information was exploited to increase the decomposition accuracy. After manually resolving differences in the discharge patterns of the same MU extracted from different channels, only one discharge pattern per MU was retained (for details, see the “Signal decomposition” section) so that each MU activity was represented by a unique discharge pattern. For automatic decomposition, all signals from the same array were decomposed with the BSS method [see Materials and Methods and (*22*)]. We then compared the MU discharge patterns extracted by the two decomposition procedures (manual and automatic) via the rate of agreement (RoA). The RoA ranges from 0 to 100%, with 0% indicating no discharge in common between two discharge patterns (within a set tolerance; see the “Assessment of the automatic decomposition accuracy” section) and 100% indicating all discharges in common.

Table 1 reports the data obtained via the decomposition process. The activity of 161 MUs was manually decomposed from the signals recorded from the four arrays, yielding 38,735 unique discharges in 20 s. The RoA between all possible pairs of MUs detected from the same array (1225, 630, 741, and 630 for S1 array 1, S1 array 2, S2, and S3, respectively) ranged from 0 to 11% (median: 2%). This value is perfectly in agreement with the expected number of synchronized discharges among MUs (*23*) and therefore indicates that all identified MU discharge patterns were unique. The number of channels in which the peak-to-peak amplitude of the corresponding action potential exceeded 10 times the root mean square (RMS) baseline noise ranged from 4 to 40 (median: 18) for all MUs but 3 (148 MUs in total). The presence of the same MU over multiple channels contributed to the accurate extraction of the MU discharge patterns (*24*). The average discharge rates were 14.8 ± 1.7 Hz (S1), 14.1 ± 1.6 Hz (S1 array 1), 15.8 ± 1.3 Hz (S1 array 2), 11.0 ± 1.2 Hz (S2), and 12.7 ± 1.9 Hz (S3), in agreement with previous studies (*25*, *26*). Most MUs were active for the whole 20-s interval, but 10 of 161 discharged less than 50 times each and were excluded from the above calculation of the average discharge rate and number of channels exceeding baseline to increase the reliability of the estimates. There were no MUs in common between array 1 and array 2 of S1 [RoA between all possible pairs (1800) ranged between 0 and 5%; median: 2%]. The cross-array spike triggered averaging procedures produced averages at the baseline noise level, further confirming that there were no MUs in common between array 1 and array 2.

**Table 1.** Decomposition performance for the high-density intramuscular signals: Manual versus automatic decomposition. PNR, pulse-to-noise ratio.

Identifier	Number of MUs (manual)	Number of MUs (automatic)	Number of MUs (common)	RoA (means ± SD, %)	Sensitivity (means ± SD, %)	Precision (means ± SD, %)	PNR (automatic, dB)
S1 array 1	50	40	39	99 ± 3	99 ± 2	99 ± 1	40.5 ± 7.4
S1 array 2	36	27	27	98 ± 4	99 ± 2	99 ± 2	41.1 ± 6.7
S2	39	27	27	100 ± 1	100 ± 1	100 ± 0	42.0 ± 5.3
S3	36	30	30	99 ± 4	99 ± 2	99 ± 2	44.9 ± 8.5

### Decomposition accuracy

Table 1 includes the comparison between the output of the manual and automatic decomposition procedures. From the 161 MUs identified by manual decomposition, 123 (76%) were identified by the automatic decomposition. Only one MU identified by automatic BSS did not match a MU extracted by manual decomposition. The investigator who performed the manual decomposition initially identified the unmatched MU, but she discarded it from further analysis because of lack of confidence in the decomposition accuracy due to the low amplitude of its action potentials. Eight of the 38 MUs (21%) that were not extracted by the automatic decomposition discharged less than 50 times.

The average RoA across the 123 MU discharge patterns that were identified by both procedures (manual and automatic) was 99 ± 3%. Of those 123 discharge patterns, 64 matched the automatic results with a 100% RoA, and another 36 had a RoA of ≥99%. We inspected the disagreement between the output of the two procedures and found that only three common MUs had a RoA in the range of 80 to 85% due to misalignments in discharge timings, which was greater than our strict threshold of 0.5 ms. One of the three MUs had a satellite action potential. The satellite potential is an action potential that follows the main one tightly linked in time (with small jitter) (*27*). Among the common MUs, 16 discharges identified by the manual decomposition and missed by the automatic decomposition were doublets. The doublet is an action potential that follows the main one with a variable interval, and it is followed by a longer-than-average interspike interval (*27*). In our study, we identified a total number of 17 doublets with time interval between the two MU action potentials constituting the doublet inferior to 30% of the average interspike interval (7.0 to 22.1 ms), according to the definition in (*28*).Together, these results indicate that the high-density intramuscular array yields high MU sampling and the activity of most of the MUs can be reliably extracted by a fully automatic procedure with comparable accuracy to manual decomposition.

The manual decomposition of each channel (20 s of recording) took >8 hours by the expert operator. The fully automatic decomposition of each array (40 channels, length of 22 s) took 2 hours and 9 min of computational time on average across the four arrays (Intel CORE i9 vPro 9Gen Processor with 32-Gb RAM).

### MU population coherence

**Fig. 2.**
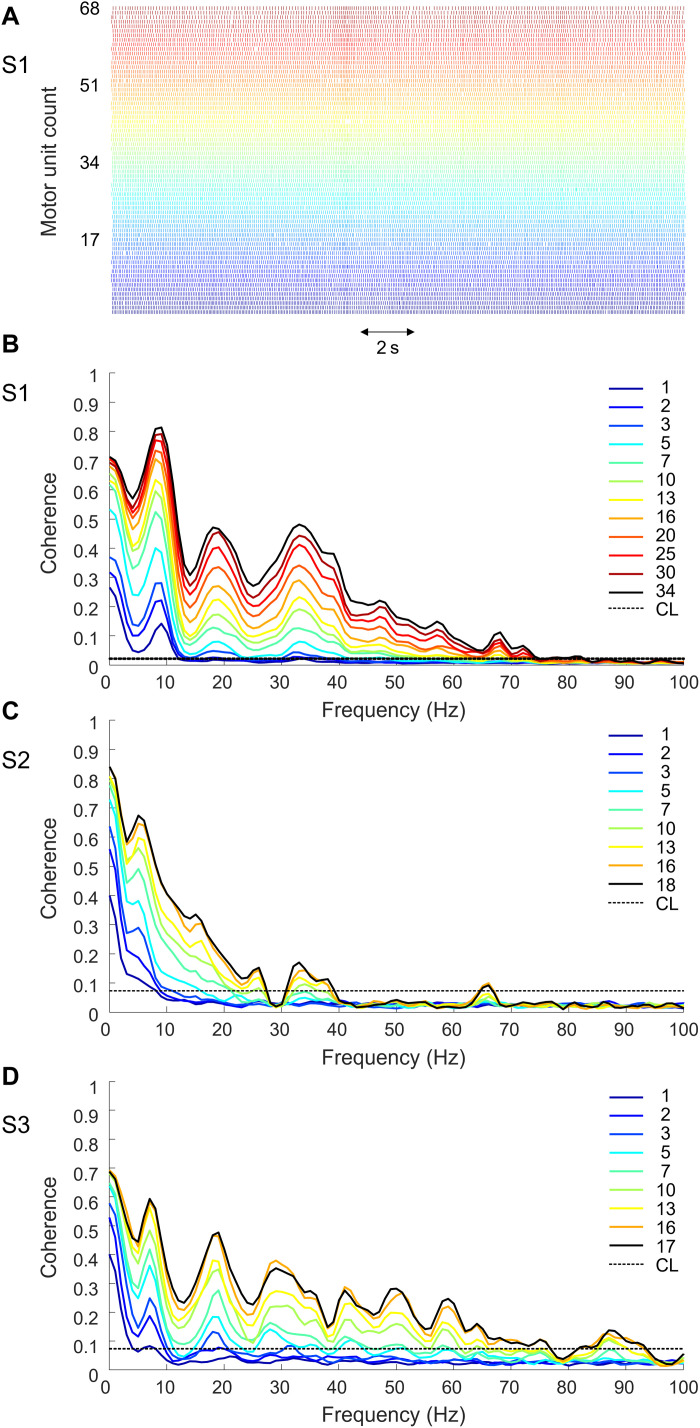
Coherence between populations of MUs. (**A**) Discharge pattern of 68 MUs active during a contraction at 20% MCV (S1, two arrays). Coherence between combinations of cumulative discharge patterns was obtained by pooling an increasing number of MUs from subjects S1 (**B**), S2 (**C**), and S3 (**D**). Black dashed horizontal line is the 95% confidence level (CL). Coherence increased with the size of the MU groups, and the population coherence was significant up to 40 Hz in S2 and up to 75 Hz in S1 and S3, respectively. Note that 60 s of data were used for S1 and 20 s for S2 and S3. Only the MUs active for whole selected interval (60 or 20 s) were included in the coherence estimation.

We calculated the coherence between groups of MU discharge patterns of increasing size (Fig. 2). Figure 2A shows 20 s of discharge patterns extracted from S1. Figure 2B shows the corresponding coherence for groups of MUs with numerosity ranging from 1 to 34 MUs. The coherence was statistically significant [i.e., above the 95% confidence level (CL)] for frequencies of up to about 75 Hz, proving that the synaptic input bandwidth goes well beyond the β band. Similarly, the coherence was still significant at ~75 Hz for S3 (Fig. 2D). In both cases, an increase of coherence in the γ band with the number of MUs is clear. On the contrary, for S2, the coherence bandwidth was limited to 40 Hz (Fig. 2C).

### Effect of MN discharges on the homonymous pool

**Fig. 3.**
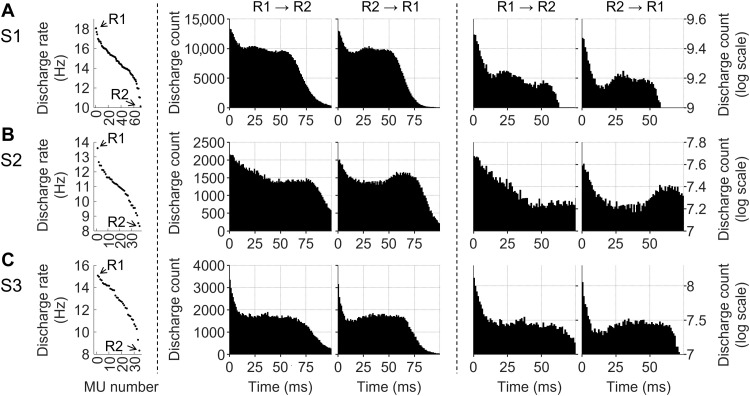
Analysis of MU synchronization. Left panels show the average discharge rate of the MUs in a 20-s time interval. Central R1 → R2 (R2 → R1) panels display the influence of MUs with higher (lower) discharge rate on the discharge timing of the MUs with lower (higher) discharge rate via pooled cross-histograms between pairs of MU discharge patterns. The two rightmost columns represent the same values in logarithmic scale so that the inhibition can be more readily visualized. The three rows represent subjects S1 (**A**), S2 (**B**), and S3 (**C**). Only the MUs active for whole selected interval (20 s) were included in the pooled cross-histogram calculation.

The discharge of a MN depends on supraspinal and spinal inputs, including from interneurons. A particular class of interneurons, the Renshaw cells, causes recurrent inhibition of the homonymous MN pool (*29*). Renshaw cells are facilitated during weak contractions and inhibited during strong contractions (*30*). We expected to see the effects of recurrent inhibition in our recordings when the subject exerted forces of 20 or 30% MVC. As there are opposing views on the distribution of recurrent inhibition between early- and late-recruited MUs within the same MN pool (*31*–*33*), we separately investigated MUs discharging at higher (R1) and lower (R2) rates. Discharge rate was considered a surrogate of recruitment order, in that early-recruited MUs discharge faster, at a given moderate level of force, than those recruited later (*34*). Results are reported in Fig. 3 as synchronization cross-histograms. As can be observed in both S1 and S3, a MU discharge inhibited the discharge of the other MUs at ~15 ms (dip in Fig. 3, A and C). On the other hand, for S2 (Fig. 3B), inhibition continued up to ~30 ms. Late-recruited MNs caused more inhibition of the discharges of the early-recruited MNs (R2 → R1, Fig. 3) than the converse (R1 → R2). The Kolmogorov-Smirnov test confirmed that the distribution of the discharge count was different (*P* < 0.05) for the bins, 9 to 19 ms (S1), 19 to 29 ms (S2), and 8 to 18 ms (S3). No dips were observed in the cross-histograms obtained by applying different perturbations (see the “Connectivity among MNs” section and figs. S1 to S3) to the original discharge patterns and maintaining the discharge rate unchanged (control condition), implying that the latter did not influence the results presented. Dips in the cross-histograms can also reflect a periodicity resulting from common drive. We calculated the output of 70 MNs receiving a common synaptic input at 33 Hz. The two cross-histograms (R1 → R2 and R2 → R1) for this simulation showed a dip at 15 to 16 ms (fig. S4), but the distribution of the discharge count in the two cases (R1 → R2 and R2 → R1) was similar according to the Kolmogorov-Smirnov test (15 ms, *P* = 0.07; 16 ms, *P* = 0.12).

## DISCUSSION

We have presented the development of a high-density electrode array for intramuscular recordings that enables the automatic accurate extraction of tens of MUs concurrently active. We have shown representative examples of MU population analysis enabled by our system.

### Intramuscular array

Our electrode array configuration consists of polymer (*35*) and metal that are micromachined (*36*) into a thread containing 40 electrodes. The materials and minimal thickness (20 μm) confer the required flexibility to interface the muscle without being unpleasant for the subject. Each electrode has an area of 5257 μm^2^. Such small electrodes inevitably present high electrical impedance, which reduces the signal-to-noise ratio. The contacts were therefore coated with microrough platinum that increases the active surface and reduces the impedance by 10 times compared to an untreated electrode (*15*, *37*). The array has electrodes manufactured on both sides of the substrate (*38*) to enable increased spatial resolution and to reduce the likelihood of short circuits. This improvement in the technology allowed us to build 40 electrodes in a 2-cm long filament.

### MU decomposition yield

Four intramuscular electrode arrays were tested in three subjects. Electrodes were inserted into the tibialis anterior and used to acquire EMG during isometric contractions at moderate force. Each array yielded an average number of 40 concurrently active MUs. Eighty-six MUs could be extracted from a contraction at 20% MVC with two high-density electrode arrays in S1. The number of MUs in the tibialis anterior has been estimated to be in the range from 122 to 445 [reviewed in (*39*)]. Therefore, given the relatively low muscle force exerted by S1, the identified 86 MUs represent a relatively large proportion of those that were active during the contraction. The number of identified discharge patterns per electrode varied in the range of 36 to 50. This variation across subjects could be related to anatomical differences, e.g., to the ratio between the muscle section covered by the electrode and the muscle thickness, which varies along the muscle, and the relative angle between the electrode and muscle fibers. Moreover, we cannot exclude a variability in the attempts to exert the maximal force, which is known to depend on the subject’s motivation (*40*, *41*). The difference in average discharge rate between array 1 and array 2 of S1 was 1.7 Hz, which was comparable to the difference between the subjects S1 and S3 (2.1 Hz), so that anatomical factors may have played the major role.

On average, 31 MUs per array could be automatically decomposed with an accuracy of 99% when compared with manual expert decomposition. Compared to previous systems with fewer electrodes (*15*), the number of automatically extracted MUs with the proposed high-density electrode is two to three times greater, and the accuracy is substantially higher (*18*). For example, our previous attempt at automatic decomposition of EMG recorded with two arrays of 16 channels each yielded 22 of 53, 24 of 57, and 21 of 60 (i.e., 42, 42, and 35%, respectively) manually detected MUs at different force levels, with an average RoA of 94%. Our high-density system enabled automatic decomposition of 76% of the manually detected MU action potential trains constituting the interference EMG with a 99% RoA. Eight MUs identified by manual decomposition discharged less than 50 times, which was insufficient for the automatic identification. The yield of MUs per channel was also superior to that achieved by BSS of high-density surface EMG data from the tibialis anterior (21 MUs per 64 channels) (*42*) that in any case can only detect MUs with large action potentials at the skin surface.

Despite the high yield, some discharge patterns identified by the manual decomposition could not be extracted automatically. BSS algorithms, as used in this study for automatic decomposition, require a number of observations (EMG channels) equal or superior to the number of sources (MUs). In this condition, theoretically, all sources can be recovered. When the number of MUs exceeds the number of channels, the automatic decomposition procedure may miss some of the sources. In this study, we used a conservative threshold on the segmentation step of the decomposition (*43*). This resulted in the accurate detection of the MU discharge patterns, although the number was inferior to the theoretical maximum. In keeping with this, it is commonly reported that BSS extracts only MUs with large action potentials at the electrode sites (highest energy) (*13*).

### MU decomposition accuracy

The automatic decomposition was validated against the manually decomposed dataset. The RoA between the two procedures was 99% on average (across 123 MUs). This value is high and can be attributed to the high density of channels. The comparison between the two decomposition procedures is a conservative approach for estimating accuracy. As signals were decomposed independently by two decomposition methods and operators, the likelihood that the same mistake is made in the two cases is very low (*24*). Therefore, the procedure of validation of the automatic decomposition in this study is robust. In addition, the average pulse-to-noise ratio across the 124 MUs automatically extracted was 42 dB (Table 1), greater than values reported for surface EMG decomposition (*44*), further confirming the high accuracy of the automatic decomposition procedure.

We inspected the disagreement between the two decomposition procedures and identified two sources of errors (doublets and misalignments). Some of the doublets could not be identified by the automatic BSS decomposition. This is to be expected, as doublets may have an action potential with different amplitude and shape compared to the main action potential (*45*). This is mainly due to the velocity recovery function of muscle fibers (*46*). The propagation of the second action potential in a doublet is facilitated by the occurrence of the first potential within a short time interval, and, therefore, the propagation velocity increases for the second potential. The change in propagation velocity depends on the interval between the two action potentials and determines a variation in the second action potential waveform (*47*). As the BSS algorithm can only identify action potentials with a similar shape, a change in amplitude/shape prevented the BSS from associating the doublet to the same MU as the main action potential. Nonetheless, an adaptive change in threshold for detection may solve this problem in the future.

Three MUs found by both decomposition procedures had misalignment for discharges of >0.5 ms, and this influenced the RoA for those MUs. These misalignments are not necessarily errors. The MU discharge pattern, detected at a certain electrode, not only produces time-locked discharge patterns in other electrodes that fall in that MU territory but can also exhibit some jitter from discharge to discharge due to fluctuations in muscle fiber conduction velocity (*48*). In retaining only one discharge pattern per MU, we discarded this information on the jitter. In addition, one of the three MUs had a satellite potential that showed some size and temporal jitter. The two algorithms may have used either the main potential or the satellite potential as a reference for the alignment, which may then cause misalignments. Note that the results of the automatic decomposition did not undergo any postprocessing. Otherwise, some mistakes could have been easily corrected by plotting the discharge rate against time to detect any inconsistencies.

This work validated the BSS decomposition on a very large number of MUs. Previous validation via comparison between surface and intramuscular data was limited to an average of 1 MU per contraction commonly found in the two datasets (*49*). In this study, rather than two datasets, we compared the decomposition performance when the same signals were independently analyzed by two operators using two different procedures. The total number of common MUs was 123, i.e., 31 per electrode array.

Automatic decomposition required much less time compared to manual decomposition. However, there were some discharge patterns identified by the manual decomposition that could not be extracted automatically. A hybrid decomposition approach could maximize the MU yield and minimize the decomposition time. The approach would consist in running a first step of automatic decomposition and then inspecting the residual signal (obtained by subtracting the template waveform of the previously identified MUs from the raw EMG signal) to complete the decomposition manually. A better option could be to extract manually only enough discharges per MU to obtain a reliable average action potential waveform and from that average to extract the filters to complete the decomposition automatically.

### MU population coherence

Our coherence analysis showed that the synaptic input common to the MN pool may have frequency content of up to 75 Hz (Fig. 2, B and D) and that the estimated coherence increases with the number of MUs included in the analysis. Therefore, large populations of concurrently active MUs are necessary to infer characteristics of the neural drive. For a certain frequency of the synaptic input to be detected as common (i.e., statistically significant in the coherence plot), the synaptic input has to be sampled at least twice as fast as that frequency component (*6*). Each MN integrates the supraspinal and afferent inputs and discharges an action potential when the net input exceeds the recruitment threshold. Under the assumption of a common input uniformly distributed to the whole MN pool (*8*), the effective sampling frequency of the synaptic input is the cumulative discharge rate of all active MNs, obtained by pooling all discharge patterns together. In voluntary sustained contractions, a MN usually discharges less than 40 action potentials per second (*7*). As a result, sampling by few MNs limits the maximal frequency of the signal recorded from the output of the spinal cord, while large populations allow the synaptic input to be reconstructed more accurately from the MN output.

The very large frequency content identified for the neural drive from the spinal cord to muscles is unexpected, as muscles can only contract within a narrow bandwidth (<10 Hz) (*50*). The issue of the mismatch between the bandwidth of the neural drive and of the muscle dynamics has been previously discussed in relation to the β band (*51*). It has long been known that β oscillations are present in MN output (*52*) while they are filtered out by the muscle contractile properties. The new observation of a much greater frequency content than the β oscillations indicates the variety of common inputs received by the MN pool. γ-range corticomuscular coherence has been observed during strong isometric voluntary contractions (*53*) and during dynamic contractions (*54*), suggesting that the γ band rhythmic drive from the cortex contributes, at least in part, to the EMG activity at that frequency band. Our results show that human muscles can manifest rhythmic electrical oscillations in the γ band also during low-intensity isometric contractions. This phenomenon is not necessarily related to the movement itself. It could be due to movement preparation and to cortical or subcortical oscillations that are transmitted to the muscle.

There was a large difference in coherence frequency content between S2 and the other two subjects. S2 and S3 performed the same task. In addition, coherence was estimated from a similar number of discharge patterns. However, we cannot exclude that the proportion of common input varied across the identified MNs at high frequencies. It is possible that we detected MNs in S2 that shared a smaller proportion of common input with respect to the MNs detected in the other subjects. Unfortunately, now, there are no methods to estimate the proportion of common input shared by MNs at high frequency, and, therefore, we cannot support this explanation directly.

### Influence of MN discharges onto the homonymous pool

Our study included the analysis of the influence of the discharges of MUs with higher discharge rates on those with lower discharge rates (R1 → R2; Fig. 3) and vice versa (R2 → R1; Fig. 3). We observed that the highest value of the six cross-histograms was obtained at 0 s, indicating the common drive received by the MN pool (*34*). MUs were less likely to discharge after about 50 (S1), 75 (S2), and 70 ms (S3). These values are inversely proportional to the respective average discharge rates for the three subjects, i.e., 14.8 (S1), 11.0 (S2), and 12.7 Hz (S3). The pooled cross-histograms decreased in values for intervals above the respective average interpulse intervals, as expected from the calculation of these values based on the discharge following the reference one (see the “Connectivity among MNs” section). MUs were less likely to fire for about 15 (S1 and S3; Fig. 3, A and C) or 30 ms (S2; Fig. 3B) after the discharge of both MUs discharging at higher (R1 → R2) or lower (R2 → R1) discharge rate. This observation is in agreement with recurrent inhibition by Renshaw cells, which occurs with similar timing (*55*). The dips in the R2 → R1 histograms were deeper than in the R1 → R2 counterpart, suggesting that MUs with lower discharge rate cause more inhibition on those with higher discharge rate than the opposite.

Recurrent inhibition has been studied in isolated cells in in vitro experiments or in anesthetized animal preparations. The main method to test homonymous recurrent inhibition in humans is indirect and relies on changes in H-reflex modulation caused by presumed recurrent effects (*56*). An elegant method to evaluate recurrent inhibition in humans at individual MN level has been proposed by Özyurt *et al*. (*57*). However, this method can only be used to assess the impact of the largest on smaller MUs, as it evaluates the effect of electrical stimulation on the background discharges of small MUs. On the contrary, our method can be applied in both directions across the MN pool during voluntary contractions. Özyurt *et al*. (*57*) reported an average latency for recurrent inhibition of 37.7 ms from a peripheral stimulus for the soleus muscle, which is compatible with the dips at ~30 ms visible in the cross-histograms of S2 (Fig. 3B). For S1 and S3, inhibition occurred earlier than for S2 (Fig. 3, A and C).

Phenomena other than recurrent inhibition may account for the dips observed in the pooled cross-histograms. The dips could have originated also from any inhibitory interneurons, which are synchronized in part with the MNs. Moreover, the cross-histograms in Fig. 3A (S1) present two dips spaced about 30 ms apart. This could reflect a periodicity with 30-ms period that may result from a common input at about 33 Hz, in agreement with the peak observed in the coherence plot in Fig. 2B at that frequency. To verify this, we simulated the output of 70 MNs that received a common synaptic input at 33 Hz and discharged at rates in the same range as S1. The common input resulted in a dip at 15 to 16 ms, followed by two dips spaced 30 ms apart, as expected [1/(30 ms) = 33 Hz]. However, the depth was statistically similar in the two cross-histograms (R1 → R2 and R1 → R2; fig. S4), while the distributions of the discharge count were different for the bins around the dip in the experimental data. While this may lean support to the hypothesis that the cross-histograms obtained from experimental data also reflect Renshaw inhibition, the common synaptic input received by the pool may have also played a role in the appearance of the dips. Future modeling and experimental work is needed to further our understanding of the interplay between common drive and inhibition. The discharge rate per se cannot be considered as the only determinant of the dips; in that the control conditions, where we simulated discharge patterns with the same discharge rate as the experimental ones, the cross-histograms did not show any dip (see the Supplementary Materials).

### Conclusions

In conclusion, we present a novel high-density intramuscular array along with a methodology that fully automatically identifies the discharge patterns of relatively large number of MUs, unveiling new knowledge behind MN population coding. We demonstrated that the number of automatically identified MUs is high enough to reveal the presence of significant coherence between groups of MNs in the frequency range of up to 75 Hz and the effect of Renshaw inhibition on the homonymous MN pool. These results constitute an important step forward in the in vivo population coding analysis of the human motor system. Future work will investigate MU behavior during dynamic contractions and high force levels.

## MATERIALS AND METHODS

### Manufacturing process

The thin-film electrode array structure was built using microfabrication processes. The electrode array was built over a silicon wafer used as a platform for the production. The structure was built layer by layer with layers of metal for tracks sandwiched between three layers of polyimide. Metals were patterned using a photolithography process.

First, a platinum etch mask was deposited and liftoff structured on a 4-inch silicon wafer. In the next step, a 5-μm polyimide layer (PI2611, HD MicroSystems) was spun on the wafer and cured at 350°C. The lower platinum electrode contacts and tracks were then sputtered and liftoff structured. Another 10-μm polyimide layer was deposited, followed by the upper platinum electrode tracks and contacts, which were sputtered and liftoff structured, followed by a final 5-μm polyimide layer for insulation. To reach the contacts on the lower side, the silicon wafer was etched from the backside using reactive ion etching. In a second reactive ion etching step, the lower electrode contacts were opened using the previously deposited platinum layer as etch mask. An aluminum etch mask was then deposited on the top side and used for reactive ion etching of the polyimide to open the contacts on the upper side. After removal of the aluminum mask, the microfabrication process was completed, and the separated double-sided electrode arrays were removed from the wafer using tweezers. The electrode contacts were coated with microrough platinum using electroplating from an aqueous solution of hexachloroplatinic acid (*58*). This reduced the electrode impedance by about one order of magnitude so that the resulting values of impedance spectroscopy were ~10 kilohms at 1 kHz. A plug (Harwin M50-4902045 connector) was soldered to the adapter as the interface with external hardware. Each electrode array was inserted into a hypodermic needle with the bevel smoothed with a laser (PICCOLASER, O.R. Lasertechnologie, DE).

### Subjects

Three healthy men (age range, 29 to 39 years) participated in the experiment, which was approved by the Ethical Committee of the University Medical Center of Göttingen and conducted according to the Declaration of Helsinki (2008).

### Experimental procedure

The subject was seated in the chair of a Biodex System 3 (Biodex Medical Systems Inc., NY, USA) with the right leg and foot stably fixated. He was asked to perform two brief MVCs with a 5-min interval in between to recover from fatigue. The peak of the two was considered as the MVC. Electrode array placement was followed by five extra minutes of rest. The skin was cleaned with alcohol. For subjects S2 and S3, one thin-film electrode array was inserted into the middle of the proximal half of the tibialis anterior muscle, perpendicular to the skin with the tip of the needle to a depth of 2.5 cm below the fat layer as estimated by ultrasound (Telemed Ltd., Vilnius, Lithuania). For subject S1, two electrode arrays were inserted at distance from each other of approximately 3 and 1 cm in the longitudinal and transversal direction of the muscle, respectively. The first array was inserted in S1 in a position 0.5 cm medial and 1.5 proximal with respect to the position of the array in subjects S2 and S3. The second array inserted in S1 was approximately 0.5 cm lateral and 1.5 distal with respect to the position of the array in S2 and S3.

Intramuscular EMG signals were recorded with a multichannel amplifier (EMG-USB2, OT Bioelettronica, Torino, Italy) with a gain of 200 to 500 and band-pass–filtered (eighth-order Bessel filter, high-pass cutoff frequency of 10 to 100 Hz and low-pass cutoff frequency of 4400 Hz), before being sampled at 10,240 Hz, using a 12-bit analog-to-digital converter. The EMG signals were acquired in a unipolar derivation with reference and ground electrodes at the ankle.

The subject was then asked to perform a brief contraction at 20 and 30% MVC during which the experimenters judged the signal quality. Following these trials, S1 was asked to perform a steady contraction at 20% MVC, whereas S2 and S3 were given 30% MVC as the target force level. Subjects were asked to perform a steady contraction lasting at least 1 min. The subject was provided with real-time force feedback displayed on a screen. The target force level was represented as straight line on the computer screen and the force exerted by the subject as a running dot. The subject was instructed to keep the position of the dot as close as possible to the straight line. He was allowed to complete the 1-min contraction at once or in multiple contractions with rest at will in between.

### Signal quality assessment

EMG signals were band-pass–filtered in the bandwidth of 100 to 4400 Hz (third-order Butterworth, zero-lag filter) so that the frequency content was the same for all signals. We quantified the baseline noise as the average across 160 channels (four electrode arrays × 40 channels per array) of the RMS of a 4-s segment of data recorded at rest.

### Signal decomposition

To assess signal decomposition, 20 s of data were selected on the basis of visual inspection of the force trace as soon as the subject reached the target force and maintained it relatively stable for a 20-s time interval. The recorded signals were independently manually and automatically decomposed into the constituent MU action potential trains by two expert investigators (S.M. and A.H., respectively). In both cases, signals were high-pass–filtered at 250 Hz before decomposition. In case of manual decomposition, intramuscular EMG signals from each thin-film array were decomposed using the decomposition software EMGLAB (*16*), which relies on spike sorting to detect MU action potentials. Each channel was decomposed independently, and the series of discharges of a single MU were manually edited for resolving missed discharges and superimpositions. This process was conducted for each MU identified from the same channel until the residual signal, obtained by subtracting all averaged MU action potentials from the raw signal, was comparable in power with the raw signal baseline noise, indicating that all MU activity had been accounted for. As the same MU could be detected in adjacent channels, the decomposition results from all channels were then merged by automatically identifying the MUs detected at more than one electrode. Discharge patterns with more than 75% discharges closer than 1 ms were considered to belong to the same MU identified on different channels. Differences in the discharge patterns of the same MU extracted from different channels were examined and resolved by the investigator in charge, so that at the final stage of the manual decomposition, each MU was represented by a unique discharge pattern.

A second investigator (A.H.) automatically decomposed the 22-s long signals (with the 20-s long interval selected for manual decomposition put in the center) using the convolution kernel compensation (CKC) algorithm (*22*). The two extra seconds were removed when comparing the discharge patterns extracted by the two different algorithms after time alignment of the common discharge patterns. To briefly summarize the algorithm working principle, assuming the absence of noise, we can express the intramuscular EMG signal *x_c_*[*k*] recorded at channel *c* as the sum of trains of action potentials (one train for each active MU)

$$x_{c}\left[k\right]=\sum_{i=1}^{M}\sum_{l=0}^{L-1}h_{ci}\left[l\right]s_{i}\left[k-l\right]\;\mathrm{with}\;s_{i}\left[k\right]=\sum_{r}\delta \left[k-\phi_{\mathrm{ir}}\right]$$

$$k=1,\ldots ,f_{S}T$$

c=1,…,N (1)

where *k* is the discrete time variable, *f*_S_ is the sampling frequency, *T* is the signal duration, *h_ci_*[*l*] is the action potential of the *i*th MU as recorded at the *c*th channel, *s_i_*[*k*] = ∑*_r_*δ[*k* − ϕ*_ir_*] is the source pulse train (discharge pattern) of the *i*th MU with discharges at times ϕ*_ir_*, *L* is the duration of the action potentials, *M* is the number of active MUs, and *N* is the number of EMG channels.

The convolutive mixture model of Eq. 1 can be rewritten as an instantaneous mixture of an extended vector of sources that includes the original sources and their delayed versions (*22*)

$$\tilde{\underline{\underline{x}}}[k]=\underline{\underline{\tilde{H}}}\;\underline{\tilde{\underline{s}}}[k]$$

with

$$\underline{\tilde{\underline{s}}}[k]=[{\tilde{s}}_{1}[k],{\tilde{s}}_{2}[k],\ldots ,{\tilde{s}}_{M}[k]{]}^{T}$$

$${\tilde{s}}_{i}[k]=[s_{i}[k],s_{i}[k-1],\ldots ,s_{i}[k-(L+R-1)]],i=1,\ldots ,M$$

$$\underline{\tilde{\underline{x}}}[k]=[{\tilde{x}}_{1}[k],{\tilde{x}}_{2}[k],\ldots ,{\tilde{x}}_{N}[k]{]}^{T}$$

$${\tilde{x}}_{c}[k]=[x_{c}[k],x_{c}[k-1],\ldots ,x_{c}[k-R]],c=1,\ldots ,N$$

$$\underline{\underline{\tilde{H}}}=\left[\begin{array}{c} {\tilde{h}}_{11}\cdots {\tilde{h}}_{1M}\\ \vdots \ddots \vdots \\ {\tilde{h}}_{N1}\cdots {\tilde{h}}_{NM} \end{array}\right]$$

$${\tilde{h}}_{ci}=\left[\begin{array}{cccccc} h_{ci}[0] & \cdots & h_{ci}[L-1] & 0 & \cdots & 0\\ 0 & \ddots & \ddots & \ddots & \ddots & \vdots \\ \vdots & \ddots & \ddots & \ddots & \ddots & 0\\ 0 & \cdots & 0 & h_{ci}[0] & \cdots & h_{ci}[L-1] \end{array}\right]$$ (2)

where *R* is the extension factor and $\${\left[\cdot \right]}^{T}\$$ denoting the transpose of a matrix.

Once the mixing matrix ($\$\underline{\underline{\tilde{H}}}\$$) is identified, the source pulse trains can be extracted by multiplying the EMG signals ($\$\underline{\underline{\tilde{x}}}\$$) by the inverse of $\$\underline{\underline{\tilde{H}}}\$$ (unmixing matrix). Note that the CKC algorithm (*22*) identifies the source pulse trains *s_i_*[*k*] in a sequential manner (i.e., one after the other), using the source deflation technique to prevent the reidentification of already identified pulse trains. The maximal number of identified pulse trains is a CKC’s parameter (*22*) and was set to 150 in our study. The estimated pulse trains are then segmented into MU discharges and the cross-talk from other active MUs. In this study, we used a segmentation algorithm that considers the discharge pattern regularity and the pulse-to-noise ratio (*43*). The pulse-to-noise ratio is a signal-based metric that has been validated to assess the decomposition accuracy of BSS-based decomposition algorithms (*44*) and has been used in this study to assess the reliability of the automatic decomposition.

### Assessment of the manual decomposition accuracy

For each electrode array (three subjects, four arrays), we report the number of MUs identified by the manual and automatic decomposition and those commonly identified by both approaches. We first inspected the results of the manual decomposition. We calculated the RoA (*49*) between each pair of MU discharge patterns identified from the same 40-channel array to ensure that they were unique. The RoA was defined as the ratio between the number of discharges that were present in both discharge patterns (common) and the sum of the number of common discharges and the number of discharges present in only one of the two discharge patterns. A tolerance of 10 sample (<1 ms) was used when identifying common discharges.

Each MU discharge pattern was accurately estimated from the comparison between the discharge patterns of that MU in multiple channels. To assess the robustness of the estimation, we calculated the multichannel MU action potentials by spike-triggered averaging (*59*), i.e., by averaging the EMG of each channel on the intervals of [−10, +10] ms around the MU discharges obtained from decomposition. For each MU, we then counted the number of channels where the peak-to-peak amplitude of the action potential was greater than 10 times the average RMS of the baseline noise across the 40 channels. The higher the number of channels exceeding the threshold, the higher the likelihood that the discharge pattern was accurately estimated (*24*).

The RoA was also used to check whether there were MUs in common between array 1 and array 2 of S1. As a further check, we performed cross-array spike-triggered averaging by averaging the EMG of each channel of array 1 (array 2) using the discharges obtained from decomposition of the EMG from array 2 (array 1) as triggers. A temporal support of 20 ms (centered about the MU discharge) was used in the spike-triggered averaging procedure to account for the propagation delay between the positions of the electrode arrays, which were about 3 cm apart. For MUs in common between the two arrays, the cross-array averaging procedure will yield an action potential with higher amplitude than the baseline noise.

### Assessment of the automatic decomposition accuracy

We then compared the MU discharge patterns extracted by the two decomposition procedures (manual and automatic) using three metrics: RoA, sensitivity, and precision. For each MU, we identified: true positive (TP) as the number of discharges identified by manual decomposition that the automatic algorithm identified within ±0.5 ms, false positive (FP) as the number of discharges identified by the automatic algorithm that did not match any manually identified discharge within ±0.5 ms, and false negative (FN) as the number of the manually identified discharges that the automatic algorithm failed to identify within ±0.5 ms. Here, RoA was defined as the ratio between the matched discharges resulting from the comparison of the two procedures and the sum of matched and unmatched discharges, which can be expressed as

$$\mathrm{RoA}=\frac{\mathrm{TP}}{\mathrm{TP}+\mathrm{FN}+\mathrm{FP}}$$ (3)

Discharge patterns with more than 75% discharges closer than 0.5 ms were considered to belong to the same MU identified by the manual and automatic procedure (common MUs).

Sensitivity and precision were defined as follows

$$\mathrm{Sensitivity}=\frac{\mathrm{TP}}{\mathrm{TP}+\mathrm{FN}}$$ (4)

$$\mathrm{Precision}=\frac{\mathrm{TP}}{\mathrm{TP}+\mathrm{FP}}$$ (5)

All three indices were expressed as a percentage, with 100% indicating the best performance.

### MU population coherence

The discriminated discharge patterns were used to compute spectral coherence between groups of MUs, with numerosity ranging from 1 to half of the maximum number of identified MUs, which was selected as the number of MUs obtained from the manual decomposition that fired for the entire duration of the selected interval (60 s for S1 and 20 s for S2 and S3). The random allocation of MUs into groups was repeated 25 times for each group size (i.e., 1, 2, 3, … MU discharge patterns), and the average coherence across the 25 repetitions was calculated. For each MU, discharge patterns were represented with binary vectors of 0 and 1 [time resolution = 1/sampling frequency = 1/(10,240 Hz)], with 1 indicating the occurrence of a discharge. Within each MU group, the discharge patterns were summed to provide a cumulative discharge pattern. Coherence analysis was performed on 0.5-s nonoverlapping Hanning windows of the cumulative discharge patterns with a length of the fast Fourier transform equal to the sampling rate. To define the significance threshold for coherence peaks, the CL was calculated as (*60*)

$$\mathrm{CL}=1-{\left(1-\alpha \right)}^{\frac{1}{N-1}}$$ (6)

where *N* and α represent the number of segments used in the coherence calculation (data length/number of windows) and the CL (95%), respectively.

### Connectivity among MNs

Connectivity among MNs was estimated by the pooled cross-histogram of the discharge of pairs of MUs (resolution of 1 ms). To consider the opposing views on the distribution of recurrent inhibition between early- and late-recruited MUs within the homonymous MN pool (*31*–*33*), we separately investigated MUs discharging at higher and lower rates. MUs were ordered by discharge rate based on the fact that, at a given force, earlier-recruited MUs discharge faster than later-recruited ones (*34*). For each subject, for each MU, and for each discharge (referred to as “reference”), we calculated the time difference between the first subsequent discharge of each of the MUs with lower (higher) discharge rate and the reference discharge to obtain the R1 → R2 (R2 → R1) pooled cross-histograms. We considered in the estimation only the MUs that discharged for the entire duration of the selected time interval (20 s). We used the Kolmogorov-Smirnov test to compare the distribution of the discharge count in the two cross-histograms (R1 → R2 versus R2 → R1). As we were only interested in comparing the dip, we applied the statistical test only to the 10 bins centered around the dip. Statistical significance was set to *P* < 0.05.

As control conditions, we generated three types of discharge patterns with the same number of discharges as the detected MUs in the same time interval and (i) uniformly distributed discharge times, (ii) equal interpulse intervals, and (iii) discharged times obtained from the experimental ones by applying a time shift of 0 to 70 ms to the whole MU action potential train (different for the different MUs, but the same for all action potentials of the same MU). These three control conditions share the same discharge rate with the original discharge patterns. Last, we simulated the output of 70 MNs (as in Fig. 3A) using an integrate-and-fire model (20 s). The peaks in the coherence plot reflect common synaptic input. For instance, in the experimental data, it is evident from Fig. 2B that the MN pool received a common synaptic input at approximately 33 Hz. To model this, we fed the integrate-and-fire MN model with a sinusoidal signal at 33 Hz and independent Gaussian noise to each neuron (control condition iv). We expect this to result in dips that are apart by 1/(33 Hz) = 30 ms.
