# Supplementary material for: Blind identification of the spinal cord output in humans with high-density electrode arrays implanted in muscles
Source: Sci Adv. 2022 Nov 16;8(46):eabo5040. doi: 10.1126/sciadv.abo5040 (PMC9668292; doi:10.1126/sciadv.abo5040)
Supplement: Supplementary file 1 — Figs. S1 to S4 [file sciadv.abo5040_sm.pdf]

Science Advances
AAAS

Supplementary Materials for

Blind identification of the spinal cord output in humans with high-density electrode arrays implanted in muscles

Silvia Muceli *et al.*

Corresponding author: Silvia Muceli, muceli@chalmers.se; Dario Farina, d.farina@imperial.ac.uk

Sci. Adv. **8**, eabo5040 (2022)
DOI: 10.1126/sciadv.abo5040

This PDF file includes:

Figs. S1 to S4

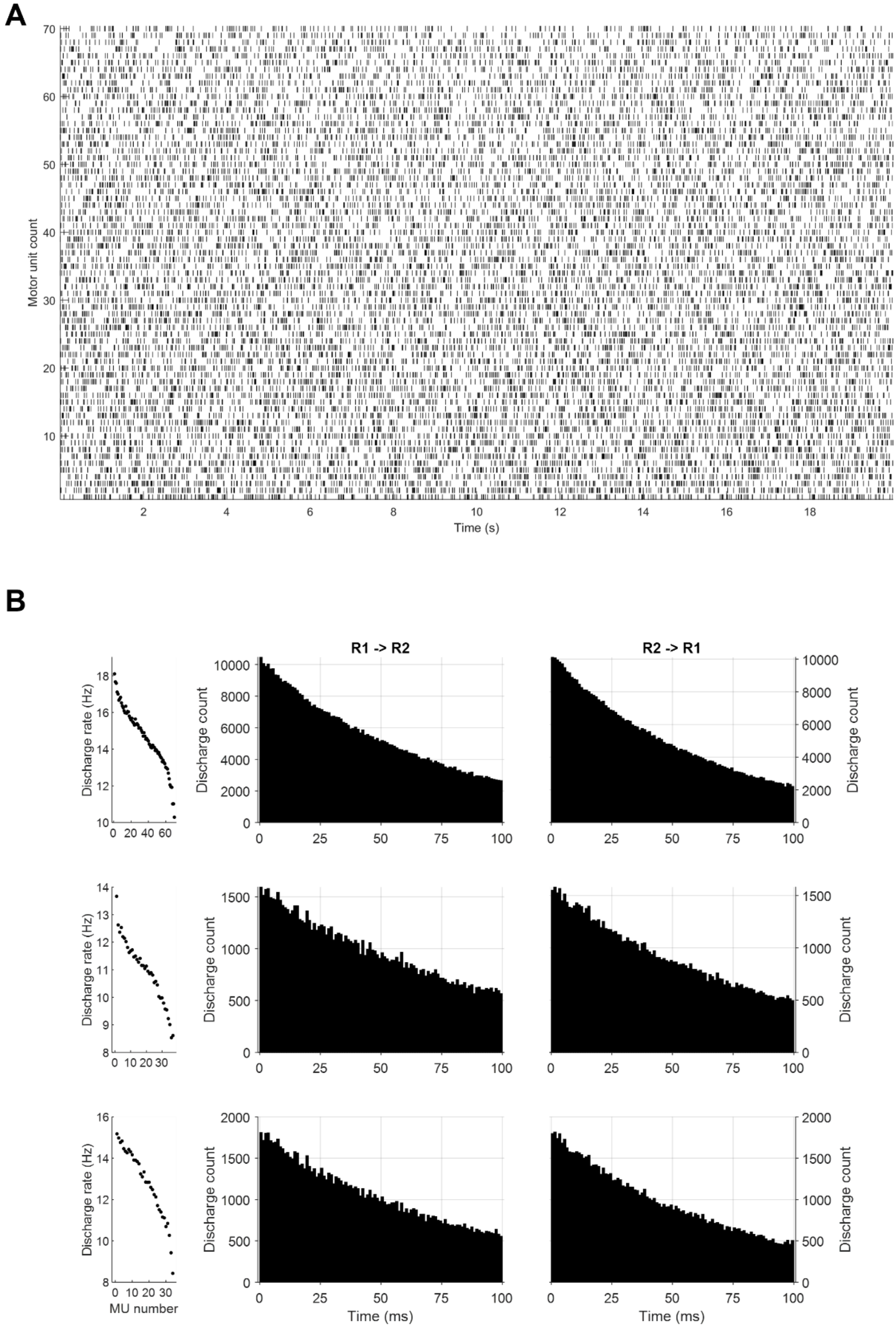
A
Motor unit count
Time (s)
B
R1 -> R2
R2 -> R1
Discharge rate (Hz)
Discharge count
MU number
Time (ms)

Fig. S1

Fig. S1.

Analysis of motor unit synchronization. A) Generated discharge patterns with uniformly distributed discharge times with the same number of discharges as the detected motor units in the same time interval in S1. B) Left panels show the average discharge rate of the motor units in a 20 s time interval. Central R1→ R2 (R2 → R1) panels display the influence of motor units with higher (lower) discharge rate on the discharge timing of the motor units with lower (higher) discharge rate via cross-histograms between pairs of motor unit discharge patterns (control condition *i)*).

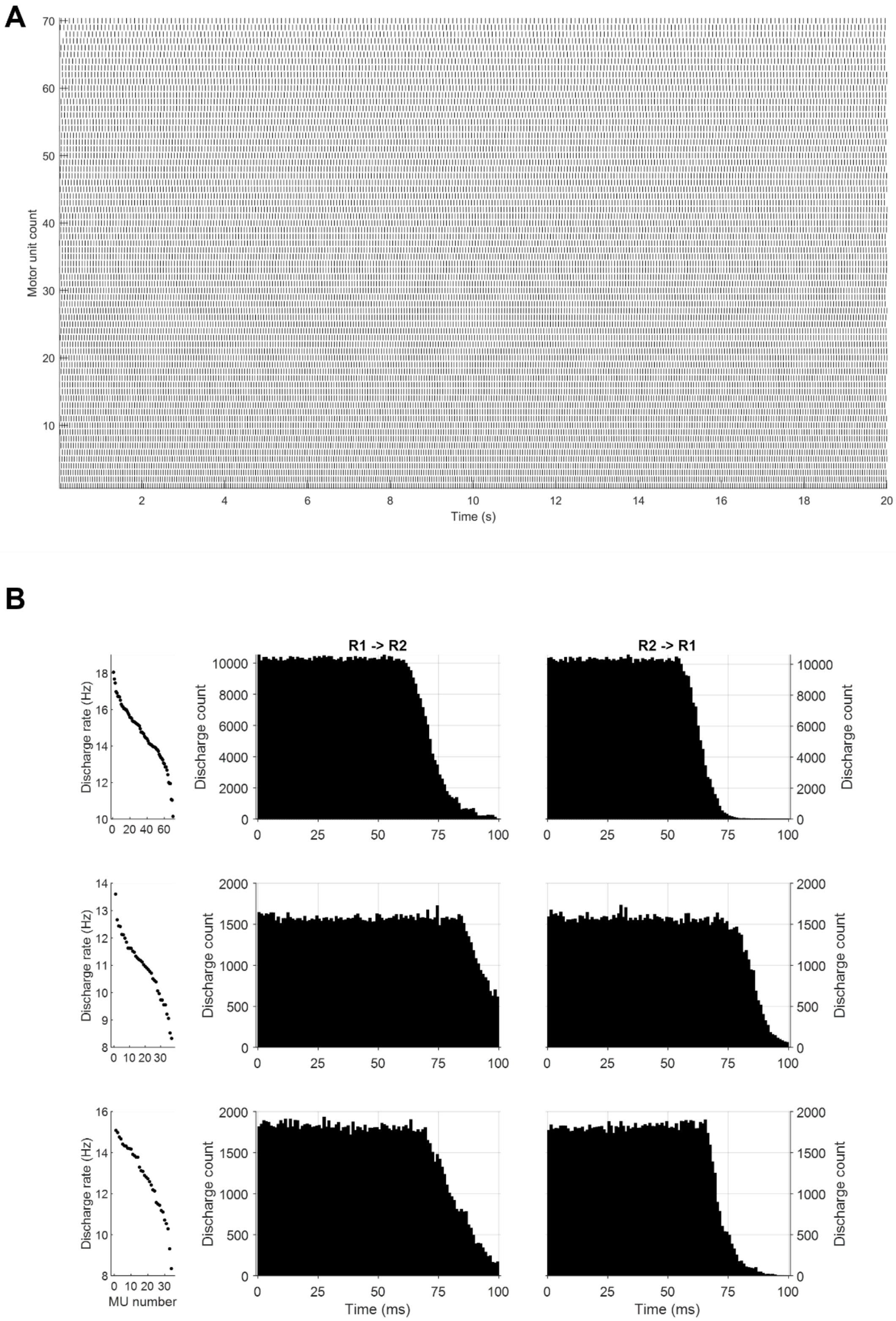
A
B
Motor unit count
Time (s)
R1 -> R2
R2 -> R1
Discharge rate (Hz)
Discharge count
MU number
Time (ms)

Fig. S2

Fig.

S2.

Analysis of motor unit synchronization. A) Generated discharge patterns with equal inter-spike intervals with the same number of discharges as the detected motor units in the same time interval in S1. B) Left panels show the average discharge rate of the motor units in a 20 s time interval. Central R1→ R2 (R2 → R1) panels display the influence of motor units with higher (lower) discharge rate on the discharge timing of the motor units with lower (higher) discharge rate via cross-histograms between pairs of motor unit discharge patterns (control condition *ii)*).

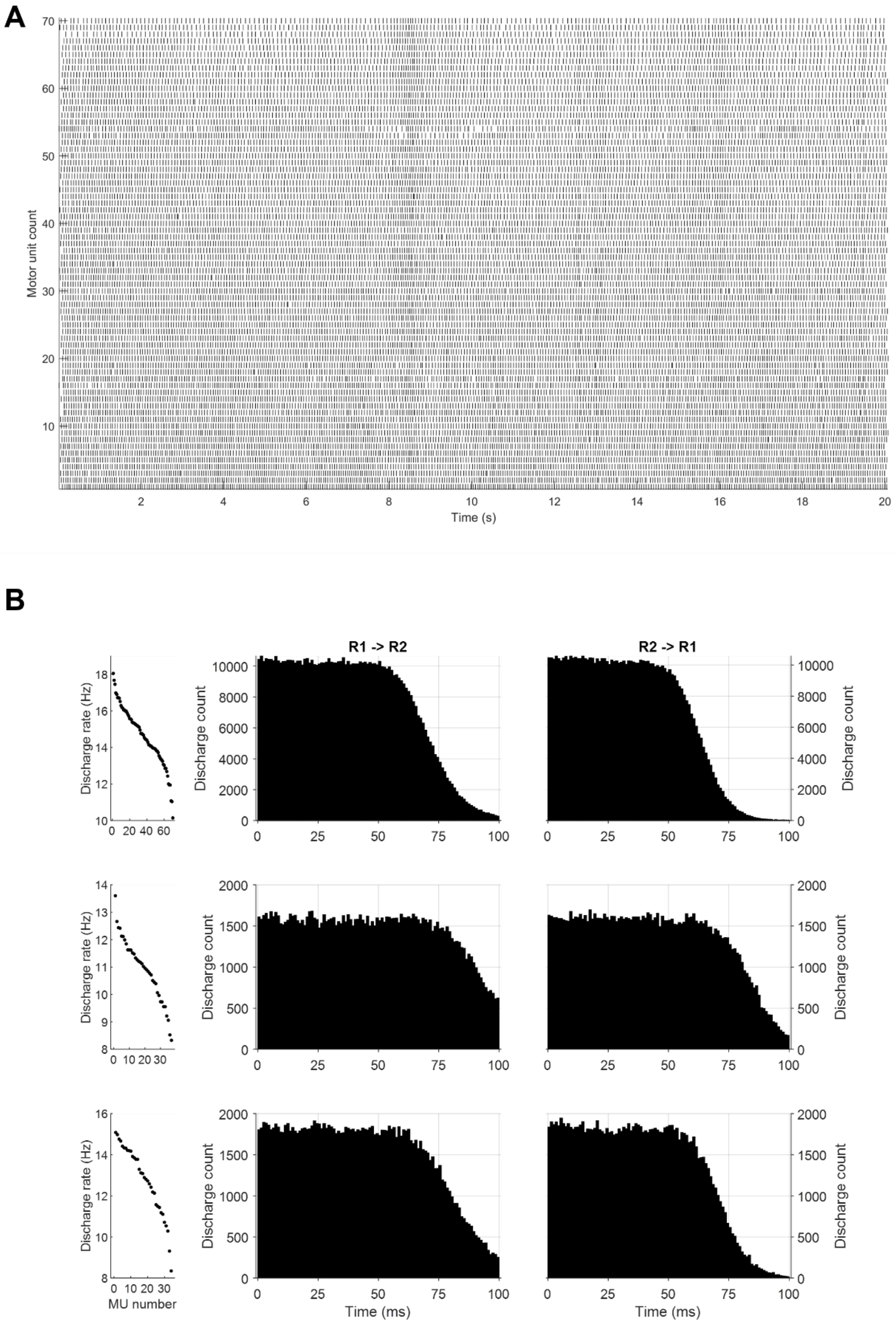
A
Motor unit count
Time (s)
B
R1 -> R2
R2 -> R1
Discharge rate (Hz)
Discharge count
MU number
Time (ms)

Fig. S3

Fig.

S3.

Analysis of motor unit synchronization. A) Discharge times generated from the experimental ones (S1) by applying a time shift of 0 to 70 ms to the whole motor unit action potential train (different for the different motor units, but the same for all action potentials of the same motor unit). B) Left panels show the average discharge rate of the motor units in a 20 s time interval. Central R1→ R2 (R2 → R1) panels display the influence of motor units with higher (lower) discharge rate on the discharge timing of the motor units with lower (higher) discharge rate via cross-histograms between pairs of motor unit discharge patterns (control condition *iii)*).

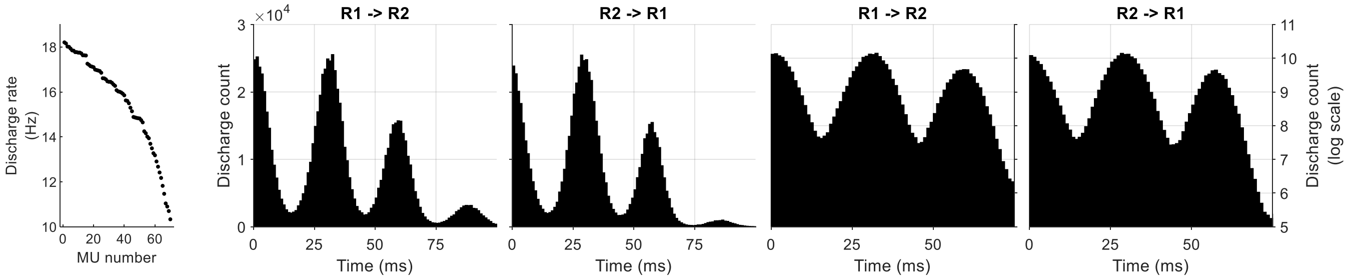

Fig S4

Fig. S4.

Analysis of motor unit synchronization. Generated discharge patterns by an integrate-and-fire model that received a common synaptic input at 33 Hz (20 s). Left panels show the average discharge rate of 70 motor units in a 20 s time interval. Central R1→ R2 (R2 → R1) panels display the influence of motor units with higher (lower) discharge rate on the discharge timing of the motor units with lower (higher) discharge rate via cross-histograms between pairs of motor unit spike trains (control condition *iv*).
